# Poly[di-μ-aqua-di­aqua­bis­(μ_7_-oxalato-κ^9^ *O* ^1^:*O* ^1^:*O* ^1^,*O* ^2^:*O* ^2^:*O* ^2′^:*O* ^2′^,*O* ^1′^:*O* ^1′^)calciumdicaesium]

**DOI:** 10.1107/S1600536813022654

**Published:** 2013-08-17

**Authors:** Hamza Kherfi, Malika Hamadène, Achoura Guehria-Laïdoudi, Slimane Dahaoui, Claude Lecomte

**Affiliations:** a Laboratoire de Cristallographie-Thermodynamique, Faculté de Chimie USTHB BP 32 El-Alia, Bab Ezzouar 16111, Alger, Algeria; b CRM2 UMR-CNRS 7036 Jean Barriol Institut, Lorraine Université BP 230, 54506 Vandoeuvre-lés-Nancy Cedex, France

## Abstract

In the title compound, [CaCs_2_(C_2_O_4_)_2_(H_2_O)_4_]_*n*_, the Ca^2+^ ion, lying on a twofold rotation axis, is coordinated by four O atoms from two oxalate ligands and two bridging water mol­ecules in an octa­hedral geometry. The Cs^+^ ion is coordinated by seven O atoms from six oxalate ligands, one bridging water and one terminal water mol­ecule. The oxalate ligand displays a scarce high denticity. The structure contains parallel chain units runnig along [10-1], formed by two edge-sharing Cs polyhedra connected by CsO_9_ polyhedra connected by a face-sharing CaO_6_ octahedron. These chains are further linked by the oxalate ligands to build up a three-dimensional framework. O—H⋯O hydrogen bonds involving the water mol­ecules and the carboxyl­ate O atoms enhance the extended structure.

## Related literature

For related compounds or structures, see: Chen *et al.* (2008 ▶); Hursthouse *et al.* (2004 ▶); Kolitsch (2004 ▶); Price *et al.* (1999 ▶); Schwendtner & Kolitsch (2004 ▶); Wu & Liu (2010 ▶).
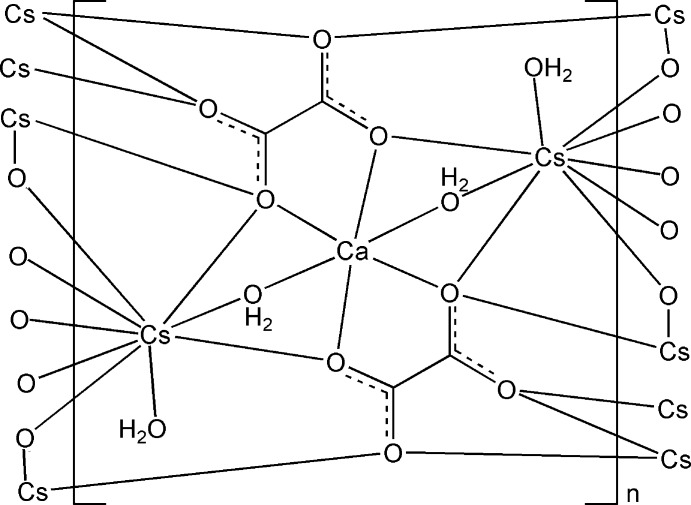


## Experimental

### 

#### Crystal data

[CaCs_2_(C_2_O_4_)_2_(H_2_O)_4_]
*M* *_r_* = 554.00
Monoclinic, 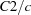

*a* = 16.8808 (4) Å
*b* = 7.3212 (2) Å
*c* = 13.5268 (3) Å
β = 128.364 (1)°
*V* = 1310.79 (6) Å^3^
*Z* = 4
Mo *K*α radiation
μ = 6.01 mm^−1^
*T* = 100 K
0.26 × 0.22 × 0.16 mm

#### Data collection

Agilent Xcalibur EOS CCD diffractometer
Absorption correction: multi-scan (*CrysAlis PRO*; Agilent, 2012 ▶) *T*_min_ = 0.229, *T*_max_ = 0.382
14337 measured reflections
2196 independent reflections
2189 reflections with *I* > 2σ(*I*)
*R*_int_ = 0.056

#### Refinement

*R*[*F* ^2^ > 2σ(*F* ^2^)] = 0.024
*wR*(*F* ^2^) = 0.060
*S* = 1.25
2196 reflections
104 parameters
6 restraints
All H-atom parameters refined
Δρ_max_ = 1.65 e Å^−3^
Δρ_min_ = −1.14 e Å^−3^

### 

Data collection: *CrysAlis PRO* (Agilent, 2012 ▶); cell refinement: *CrysAlis PRO*; data reduction: *CrysAlis PRO*; program(s) used to solve structure: *SHELXS97* (Sheldrick, 2008 ▶); program(s) used to refine structure: *SHELXL97* (Sheldrick, 2008 ▶); molecular graphics: *ORTEP-3 for Windows* (Farrugia, 2012 ▶) and *DIAMOND* (Brandenburg, 1999 ▶); software used to prepare material for publication: *WinGX* (Farrugia, 2012 ▶).

## Supplementary Material

Crystal structure: contains datablock(s) I. DOI: 10.1107/S1600536813022654/hy2635sup1.cif

Structure factors: contains datablock(s) I. DOI: 10.1107/S1600536813022654/hy2635Isup2.hkl

Additional supplementary materials:  crystallographic information; 3D view; checkCIF report

## Figures and Tables

**Table 1:** Hydrogen-bond geometry (Å, °)

*D*—H⋯*A*	*D*—H	H⋯*A*	*D*⋯*A*	*D*—H⋯*A*
O1*W*—H1⋯O2^i^	0.81 (7)	1.91 (7)	2.714 (4)	172 (6)
O1*W*—H2⋯O3^ii^	0.81 (4)	1.95 (4)	2.736 (2)	163 (7)
O2*W*—H3⋯O1*W* ^iii^	0.82 (3)	1.89 (3)	2.680 (2)	164 (5)
O2*W*—H4⋯O4^iv^	0.81 (4)	1.92 (3)	2.724 (3)	176 (7)

Symmetry codes: (i) 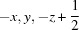; (ii) 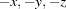; (iii) 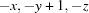; (iv) 
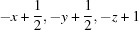
.

**Fig. 1.**
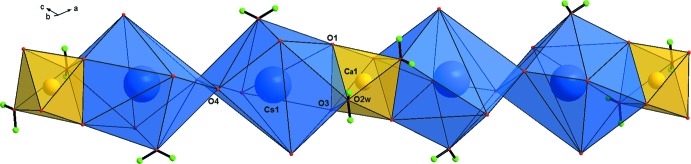
The chain built of Cs polyhedra and Ca octahedra.

**Fig. 2.**
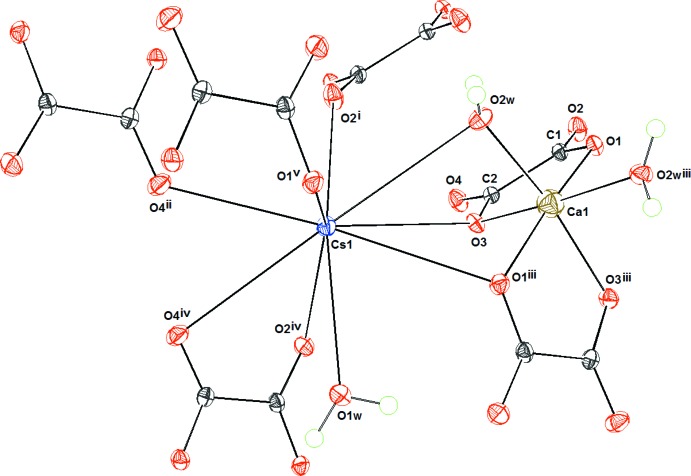
The metal atom environments of the title compound. Displacement ellipsoids are drawn at the 50% probability level. [Symmetry codes: (i) -x+1/2, -y+1/2, -z+1; (ii) -x+1/2, y+1/2, -z+1/2; (iii) -x, y, -z+1/2; (iv) x, -y, z-1/2; (v) x, -y+1, z-1/2.]

**Fig. 3.**
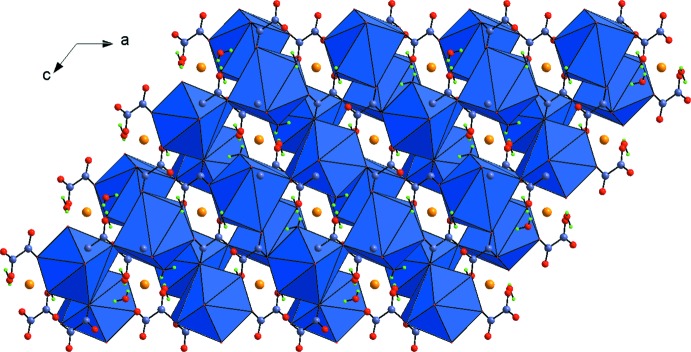
The Cs polyhedra layer showing pseudo-hexagonal cavities.

**Fig. 4.**
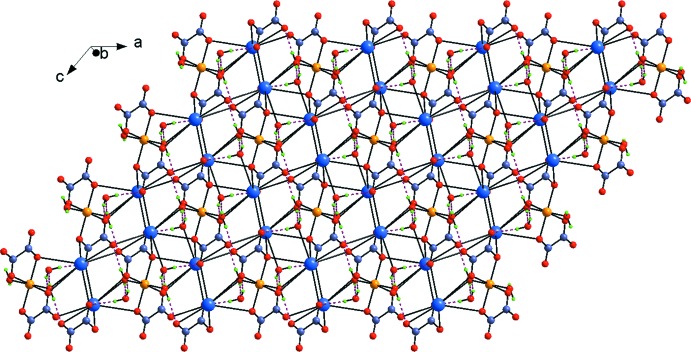
The three-dimensional packing structure showing hydrogen bonds as dashed lines.

## supplementary crystallographic information

### 1. Comment

In oxalates, A*_x_*M(C_2_O_4_)_2_(H_2_O)_n_, containing alkaline A, and alkaline-earth *M*, the A–Mg oxalates (A = Na; Cs) are isostructural with Co(II) analogue (Schwendtner & Kolitsch, 2004), owing to the corresponding ionic radii which are approximately equal [0.65 and 0.72 Å for Co(II) and Mg(II), respectively]. However, the single crystals are not easily obtained, and their growths require alternative reagents, which are not incorporated in the structure (Chen *et al.*, 2008; Kolitsch, 2004). It is the case for the title bi-metallic Cs–Ca compound, where Cr(III) oxide used in synthesis mixture does not react but serves probably in process growth as enhancing inclusion. The same specific single-crystal synthesis conditions are noticed in a mono-metallic Sr oxalate, which needs divalent transition metal to favour single crystals growth (Price *et al.*, 1999). The title compound, investigated at 100 K, is isostructural with the Mg(II) analogue (Kolitsch, 2004) and the Co(II) analogue (Schwendtner & Kolitsch, 2004) previously studied at room temperature. In the title compound, the binuclear Cs units, formed by two edge-sharing Cs polyhedra, are connected by a face-sharing Ca octahedron runnig along [101] (Fig. 1), with a nearly linear Cs—Ca—Cs and Cs—Cs—Ca chain [175.41 (6) and 172.26 (8)°, respectively]. The Cs and Ca ions have the smallest separation of 4.0528 (4) Å, whereas the longest distance [5.6889 (5) Å] occurs between the adjacent Cs atoms bridged by O4 atoms. As shown in Fig. 2, the alkaline atom is nona-coordinated by seven O atoms from six oxalate ligands, one bridging water and one terminal water molecule, while the alkaline-earth atom, which is located on a twofold rotation axis, is bound to two equivalent oxalate groups and two water molecules, the later being in a *cis* arrangement (Fig. 2). In the CaO_4_(H_2_O)_2_ octahedron, the organic ligands are coordinate with the metal centre in an expected η^4^ chelation, observed usually with this rigid ligand. The two resulting five-membered rings are perpendicular, the dihedral angle being of 89.76 (6)°. The equatorial plane is defined by two equivalent aqua ligands in *cis* position and two equivalent O3 atoms, while the axial positions are occupied by O1 atoms. The maximum atomic deviation from the mean plane [O2w, Ca1, O3, O3^iii^, O2w^iii^; symmetry code: (iii) -x, y, -z+1/2] is 0.165 (2) Å for the O3 donor. In connecting the Cs and Ca polyhedra, the oxalate ligand acts as bis-chelate, forming five-menbered rings with the both cations. Moreover, it bridges the metal atoms *via* all its O atoms, and is surrounded by six Cs and one Ca atoms through the two carboxylate groups. One group (O1, C1, O2) bridges five atoms (4 Cs and 1 Ca) and adopts an unusual µ_5_:η^3^-η^2^ coordination mode; the other group (O3, C2, O4) bridges four atoms (3 Cs and 1 Ca) and adopts a common µ_4_:η^2^-η^2^ coordination mode. As a result, the oxalate dianion displays an interesting nonadenticity in its chelating and bridging coordination modes, involving three triply bonding O atoms (O2, O3 and O4) and a tetrabonding one (O1). In the overall packing, the Cs polyhedra, linked alternately by one edge (O1···O1) and one corner (O2 atom) in a one-dimensional ladder running approximately along the [110] direction, are interconnected by the vertices to generate a two-dimensional network drawing small hexagonal cavities (Fig. 3). The adjacent pseudo-layers thus obtained, are stacked along [101] through O4···O4 edges, to give rise to a dense three-dimensional framework. Water molecules provide links between the both metallic atoms. O—H···O hydrogen bonds involving the water molecules and the carboxylate O atoms enhance the extended structure (Table 1, Fig. 4).

### 2. Experimental

The synthetic preparation was carried out in an aqueous solution at room temperature. The starting materials, caesium carbonate, trivalent chromium oxide and oxalic acid dihydrate, in equal stoechiometric amounts (0.5 mmol) were dissolved in deionized water (20 ml) and the resulting dark pink solution was stirred for two hours. The title compound crystallizes from the slowly evaporated solution after about one month and then filtered from chromium oxide powder. A few light pink crystals with prismatic geometry and suitable size for X-ray analysis were found trapped in colourless crystals of oxalic acid, then isolated, filtered and washed with diethyl ether. The light pink colour, which can not be removed after several washing, shows that small Cr(III) inclusion is present but not incorporated in the structure.

### 3. Refinement

The H atoms were located in a difference Fourier map and were refined isotropically. The highest electron density in the final difference Fourier map is 0.76 Å from Cs1 and the deepest hole is 0.72 Å from Cs1.

### Crystal data

**Table:**

[CaCs_2_(C_2_O_4_)_2_(H_2_O)_4_]	*F*(000) = 1032
*M**_r_* = 554.00	*D*_x_ = 2.807 Mg m^−^^3^
Monoclinic, *C*2/*c*	Mo *K*α radiation, λ = 0.71073 Å
Hall symbol: -C 2yc	Cell parameters from 1722 reflections
*a* = 16.8808 (4) Å	θ = 1.9–32.0°
*b* = 7.3212 (2) Å	µ = 6.01 mm^−^^1^
*c* = 13.5268 (3) Å	*T* = 100 K
β = 128.364 (1)°	Prismatic, pink
*V* = 1310.79 (6) Å^3^	0.26 × 0.22 × 0.16 mm
*Z* = 4	

### Data collection

**Table:**

Agilent Xcalibur EOS CCD diffractometer	2196 independent reflections
Radiation source: fine-focus sealed tube	2189 reflections with *I* > 2σ(*I*)
Graphite monochromator	*R*_int_ = 0.056
w scans	θ_max_ = 32.0°, θ_min_ = 3.1°
Absorption correction: multi-scan (*CrysAlis PRO*; Agilent, 2012)	*h* = −25→25
*T*_min_ = 0.229, *T*_max_ = 0.382	*k* = −10→10
14337 measured reflections	*l* = −20→20

### Refinement

**Table:**

Refinement on *F*^2^	Secondary atom site location: difference Fourier map
Least-squares matrix: full	Hydrogen site location: inferred from neighbouring sites
*R*[*F*^2^ > 2σ(*F*^2^)] = 0.024	All H-atom parameters refined
*wR*(*F*^2^) = 0.060	*w* = 1/[σ^2^(*F*_o_^2^) + (0.0199*P*)^2^ + 5.1138*P*] where *P* = (*F*_o_^2^ + 2*F*_c_^2^)/3
*S* = 1.25	(Δ/σ)_max_ < 0.001
2196 reflections	Δρ_max_ = 1.65 e Å^−^^3^
104 parameters	Δρ_min_ = −1.14 e Å^−^^3^
6 restraints	Extinction correction: *SHELXL97* (Sheldrick, 2008), Fc^*^=kFc[1+0.001xFc^2^λ^3^/sin(2θ)]^-1/4^
Primary atom site location: structure-invariant direct methods	Extinction coefficient: 0.0048 (4)

### Special details

**Table:**

Geometry. All e.s.d.'s (except the e.s.d. in the dihedral angle between two l.s. planes) are estimated using the full covariance matrix. The cell e.s.d.'s are taken into account individually in the estimation of e.s.d.'s in distances, angles and torsion angles; correlations between e.s.d.'s in cell parameters are only used when they are defined by crystal symmetry. An approximate (isotropic) treatment of cell e.s.d.'s is used for estimating e.s.d.'s involving l.s. planes.
Refinement. Refinement of *F*^2^ against ALL reflections. The weighted *R*-factor *wR* and goodness of fit *S* are based on *F*^2^, conventional *R*-factors *R* are based on *F*, with *F* set to zero for negative *F*^2^. The threshold expression of *F*^2^ > σ(*F*^2^) is used only for calculating *R*-factors(gt) *etc*. and is not relevant to the choice of reflections for refinement. *R*-factors based on *F*^2^ are statistically about twice as large as those based on *F*, and *R*- factors based on ALL data will be even larger.

### Fractional atomic coordinates and isotropic or equivalent isotropic displacement parameters (Å^2^)

**Table:**

	*x*	*y*	*z*	*U*_iso_*/*U*_eq_	
Cs1	0.152844 (10)	0.314498 (19)	0.109026 (12)	0.01416 (8)	
Ca1	0.0000	0.33665 (10)	0.2500	0.01801 (14)	
C1	0.12874 (17)	0.1846 (3)	0.4980 (2)	0.0118 (4)	
O1	0.06123 (14)	0.3069 (2)	0.43831 (17)	0.0135 (3)	
O2	0.17036 (14)	0.1342 (3)	0.60819 (16)	0.0183 (3)	
C2	0.15913 (16)	0.0871 (3)	0.4234 (2)	0.0114 (3)	
O4	0.22599 (14)	−0.0311 (3)	0.47650 (17)	0.0179 (3)	
O3	0.10886 (13)	0.1373 (2)	0.30886 (16)	0.0133 (3)	
O1W	−0.03349 (15)	0.1582 (2)	−0.15099 (18)	0.0161 (3)	
H1	−0.071 (3)	0.159 (6)	−0.133 (5)	0.033 (12)*	
H2	−0.049 (4)	0.079 (5)	−0.202 (4)	0.037 (12)*	
O2W	0.10155 (13)	0.5272 (2)	0.28003 (17)	0.0162 (3)	
H3	0.088 (4)	0.632 (4)	0.254 (4)	0.027 (10)*	
H4	0.154 (3)	0.532 (8)	0.351 (3)	0.050 (15)*	

### Atomic displacement parameters (Å^2^)

**Table:**

	*U*^11^	*U*^22^	*U*^33^	*U*^12^	*U*^13^	*U*^23^
Cs1	0.01329 (9)	0.01645 (10)	0.01254 (9)	−0.00098 (4)	0.00792 (7)	−0.00063 (4)
Ca1	0.0185 (3)	0.0180 (3)	0.0172 (3)	0.000	0.0109 (3)	0.000
C1	0.0101 (8)	0.0146 (9)	0.0100 (9)	−0.0015 (6)	0.0060 (7)	−0.0013 (6)
O1	0.0136 (7)	0.0154 (7)	0.0114 (7)	0.0021 (5)	0.0077 (6)	−0.0001 (5)
O2	0.0168 (7)	0.0263 (9)	0.0110 (7)	0.0042 (7)	0.0082 (6)	0.0034 (6)
C2	0.0111 (8)	0.0115 (8)	0.0113 (8)	−0.0003 (7)	0.0068 (7)	−0.0013 (6)
O4	0.0160 (7)	0.0200 (8)	0.0154 (7)	0.0066 (6)	0.0086 (6)	0.0012 (6)
O3	0.0151 (7)	0.0138 (7)	0.0110 (7)	0.0018 (6)	0.0082 (6)	0.0005 (5)
O1W	0.0188 (8)	0.0156 (7)	0.0158 (8)	−0.0026 (6)	0.0117 (7)	−0.0031 (6)
O2W	0.0132 (7)	0.0142 (7)	0.0154 (7)	−0.0014 (6)	0.0060 (6)	0.0022 (6)

### Geometric parameters (Å, º)

**Table:**

Cs1—O2^i^	3.0837 (18)	Ca1—O1	2.0823 (18)
Cs1—O4^ii^	3.1206 (18)	C1—O2	1.246 (3)
Cs1—O1W	3.1369 (19)	C1—O1	1.269 (3)
Cs1—O1^iii^	3.2557 (18)	C1—C2	1.559 (3)
Cs1—O2^iv^	3.299 (2)	C2—O4	1.238 (3)
Cs1—O1^v^	3.3124 (17)	C2—O3	1.275 (3)
Cs1—O2W	3.318 (2)	O1W—H1	0.81 (3)
Cs1—O4^iv^	3.434 (2)	O1W—H2	0.81 (3)
Cs1—O3	3.4688 (17)	O2W—H3	0.82 (3)
Ca1—O2W	2.0456 (18)	O2W—H4	0.81 (3)
Ca1—O3	2.0797 (18)		
			
O2^i^—Cs1—O4^ii^	93.63 (5)	O3^iii^—Ca1—C2^iii^	23.63 (6)
O2^i^—Cs1—O1W	163.78 (5)	O3—Ca1—C2^iii^	91.29 (7)
O4^ii^—Cs1—O1W	98.53 (5)	O1—Ca1—C2^iii^	115.82 (7)
O2^i^—Cs1—O1^iii^	110.34 (5)	O1^iii^—Ca1—C2^iii^	55.35 (6)
O4^ii^—Cs1—O1^iii^	146.74 (5)	C1^iii^—Ca1—C2^iii^	31.60 (6)
O1W—Cs1—O1^iii^	63.58 (5)	C1—Ca1—C2^iii^	110.84 (6)
O2^i^—Cs1—O2^iv^	96.06 (5)	O2W^iii^—Ca1—C2	147.17 (7)
O4^ii^—Cs1—O2^iv^	106.19 (5)	O2W—Ca1—C2	91.60 (6)
O1W—Cs1—O2^iv^	70.28 (5)	O3^iii^—Ca1—C2	91.29 (7)
O1^iii^—Cs1—O2^iv^	94.20 (5)	O3—Ca1—C2	23.63 (6)
O2^i^—Cs1—O1^v^	115.89 (5)	O1—Ca1—C2	55.35 (6)
O4^ii^—Cs1—O1^v^	64.42 (5)	O1^iii^—Ca1—C2	115.82 (7)
O1W—Cs1—O1^v^	79.29 (5)	C1^iii^—Ca1—C2	110.84 (6)
O1^iii^—Cs1—O1^v^	84.04 (4)	C1—Ca1—C2	31.60 (6)
O2^iv^—Cs1—O1^v^	146.60 (4)	C2^iii^—Ca1—C2	100.91 (9)
O2^i^—Cs1—O2W	63.16 (5)	O2W^iii^—Ca1—Cs1^iii^	54.62 (5)
O4^ii^—Cs1—O2W	127.09 (5)	O2W—Ca1—Cs1^iii^	129.31 (6)
O1W—Cs1—O2W	116.03 (5)	O3^iii^—Ca1—Cs1^iii^	58.85 (5)
O1^iii^—Cs1—O2W	53.63 (4)	O3—Ca1—Cs1^iii^	117.46 (5)
O2^iv^—Cs1—O2W	122.16 (4)	O1—Ca1—Cs1^iii^	52.98 (5)
O1^v^—Cs1—O2W	83.25 (4)	O1^iii^—Ca1—Cs1^iii^	126.43 (5)
O2^i^—Cs1—O4^iv^	109.66 (5)	C1^iii^—Ca1—Cs1^iii^	107.11 (5)
O4^ii^—Cs1—O4^iv^	59.65 (6)	C1—Ca1—Cs1^iii^	71.01 (5)
O1W—Cs1—O4^iv^	68.25 (5)	C2^iii^—Ca1—Cs1^iii^	79.22 (4)
O1^iii^—Cs1—O4^iv^	127.01 (4)	C2—Ca1—Cs1^iii^	97.83 (4)
O2^iv^—Cs1—O4^iv^	48.27 (4)	O2W^iii^—Ca1—Cs1	129.31 (6)
O1^v^—Cs1—O4^iv^	107.77 (4)	O2W—Ca1—Cs1	54.62 (5)
O2W—Cs1—O4^iv^	168.93 (4)	O3^iii^—Ca1—Cs1	117.46 (5)
O2^i^—Cs1—O3	65.75 (5)	O3—Ca1—Cs1	58.85 (5)
O4^ii^—Cs1—O3	158.59 (4)	O1—Ca1—Cs1	126.43 (5)
O1W—Cs1—O3	100.76 (5)	O1^iii^—Ca1—Cs1	52.98 (5)
O1^iii^—Cs1—O3	53.03 (4)	C1^iii^—Ca1—Cs1	71.01 (5)
O2^iv^—Cs1—O3	72.14 (4)	C1—Ca1—Cs1	107.11 (5)
O1^v^—Cs1—O3	128.54 (4)	C2^iii^—Ca1—Cs1	97.83 (4)
O2W—Cs1—O3	50.03 (4)	C2—Ca1—Cs1	79.22 (4)
O4^iv^—Cs1—O3	120.07 (4)	Cs1^iii^—Ca1—Cs1	175.41 (2)
O2^i^—Cs1—C1^iv^	112.56 (5)	O2W^iii^—Ca1—H3	80.6 (9)
O4^ii^—Cs1—C1^iv^	97.66 (5)	O2W—Ca1—H3	14.7 (9)
O1W—Cs1—C1^iv^	55.28 (5)	O3^iii^—Ca1—H3	161.3 (9)
O1^iii^—Cs1—C1^iv^	94.08 (4)	O3—Ca1—H3	102.5 (9)
O2^iv^—Cs1—C1^iv^	17.78 (4)	O1—Ca1—H3	102.7 (10)
O1^v^—Cs1—C1^iv^	128.86 (4)	O1^iii^—Ca1—H3	87.3 (10)
O2W—Cs1—C1^iv^	134.64 (5)	C1^iii^—Ca1—H3	110.7 (10)
O4^iv^—Cs1—C1^iv^	38.09 (4)	C1—Ca1—H3	107.0 (10)
O3—Cs1—C1^iv^	85.85 (4)	C2^iii^—Ca1—H3	141.0 (10)
O2^i^—Cs1—C1^v^	98.81 (5)	C2—Ca1—H3	106.2 (9)
O4^ii^—Cs1—C1^v^	56.66 (5)	Cs1^iii^—Ca1—H3	123.2 (10)
O1W—Cs1—C1^v^	96.84 (5)	Cs1—Ca1—H3	61.3 (10)
O1^iii^—Cs1—C1^v^	95.90 (5)	O2W^iii^—Ca1—H4	100.2 (12)
O2^iv^—Cs1—C1^v^	157.84 (5)	O2W—Ca1—H4	16.9 (10)
O1^v^—Cs1—C1^v^	18.14 (4)	O3^iii^—Ca1—H4	169.1 (11)
O2W—Cs1—C1^v^	79.44 (5)	O3—Ca1—H4	79.6 (12)
O4^iv^—Cs1—C1^v^	110.71 (4)	O1—Ca1—H4	80.4 (10)
O3—Cs1—C1^v^	129.22 (4)	O1^iii^—Ca1—H4	106.6 (10)
C1^iv^—Cs1—C1^v^	141.18 (6)	C1^iii^—Ca1—H4	129.7 (10)
O2^i^—Cs1—C2^iv^	119.80 (5)	C1—Ca1—H4	78.8 (10)
O4^ii^—Cs1—C2^iv^	75.48 (5)	C2^iii^—Ca1—H4	159.8 (11)
O1W—Cs1—C2^iv^	54.11 (5)	C2—Ca1—H4	77.8 (11)
O1^iii^—Cs1—C2^iv^	109.27 (4)	Cs1^iii^—Ca1—H4	121.0 (11)
O2^iv^—Cs1—C2^iv^	37.89 (4)	Cs1—Ca1—H4	62.0 (11)
O1^v^—Cs1—C2^iv^	111.62 (4)	H3—Ca1—H4	29.4 (14)
O2W—Cs1—C2^iv^	157.40 (4)	O2—C1—O1	125.9 (2)
O4^iv^—Cs1—C2^iv^	17.93 (4)	O2—C1—C2	118.1 (2)
O3—Cs1—C2^iv^	108.86 (4)	O1—C1—C2	116.0 (2)
C1^iv^—Cs1—C2^iv^	23.10 (5)	O2—C1—Ca1	166.71 (17)
C1^v^—Cs1—C2^iv^	119.95 (4)	C2—C1—Ca1	74.64 (12)
O2^i^—Cs1—H1	154.3 (7)	O2—C1—Cs1^vi^	53.94 (13)
O4^ii^—Cs1—H1	111.6 (6)	O1—C1—Cs1^vi^	138.48 (15)
O1W—Cs1—H1	14.1 (5)	C2—C1—Cs1^vi^	79.72 (11)
O1^iii^—Cs1—H1	49.6 (5)	Ca1—C1—Cs1^vi^	130.00 (7)
O2^iv^—Cs1—H1	72.7 (8)	O2—C1—Cs1^vii^	88.32 (14)
O1^v^—Cs1—H1	81.2 (8)	O1—C1—Cs1^vii^	54.36 (11)
O2W—Cs1—H1	102.6 (6)	C2—C1—Cs1^vii^	133.18 (13)
O4^iv^—Cs1—H1	80.5 (6)	Ca1—C1—Cs1^vii^	84.67 (5)
O3—Cs1—H1	88.6 (6)	Cs1^vi^—C1—Cs1^vii^	141.18 (6)
C1^iv^—Cs1—H1	60.7 (8)	C1—O1—Ca1	114.80 (15)
C1^v^—Cs1—H1	99.3 (8)	C1—O1—Cs1^iii^	125.89 (14)
C2^iv^—Cs1—H1	64.9 (6)	Ca1—O1—Cs1^iii^	96.32 (6)
O2W^iii^—Ca1—O2W	94.03 (11)	C1—O1—Cs1^vii^	107.50 (14)
O2W^iii^—Ca1—O3^iii^	88.28 (7)	Ca1—O1—Cs1^vii^	115.34 (7)
O2W—Ca1—O3^iii^	170.75 (7)	Cs1^iii^—O1—Cs1^vii^	95.96 (4)
O2W^iii^—Ca1—O3	170.75 (7)	C1—O2—Cs1^i^	147.27 (17)
O2W—Ca1—O3	88.28 (7)	C1—O2—Cs1^vi^	108.28 (15)
O3^iii^—Ca1—O3	90.86 (10)	Cs1^i^—O2—Cs1^vi^	97.86 (5)
O2W^iii^—Ca1—O1	91.87 (7)	O4—C2—O3	125.9 (2)
O2W—Ca1—O1	96.30 (7)	O4—C2—C1	119.50 (19)
O3^iii^—Ca1—O1	92.57 (7)	O3—C2—C1	114.60 (18)
O3—Ca1—O1	78.97 (7)	O4—C2—Ca1	166.69 (16)
O2W^iii^—Ca1—O1^iii^	96.30 (7)	C1—C2—Ca1	73.76 (11)
O2W—Ca1—O1^iii^	91.87 (7)	O4—C2—Cs1^vi^	58.69 (13)
O3^iii^—Ca1—O1^iii^	78.97 (7)	O3—C2—Cs1^vi^	137.27 (14)
O3—Ca1—O1^iii^	92.57 (7)	C1—C2—Cs1^vi^	77.18 (11)
O1—Ca1—O1^iii^	168.01 (10)	Ca1—C2—Cs1^vi^	128.24 (7)
O2W^iii^—Ca1—C1^iii^	95.70 (7)	C2—O4—Cs1^viii^	127.57 (15)
O2W—Ca1—C1^iii^	115.59 (7)	C2—O4—Cs1^vi^	103.37 (14)
O3^iii^—Ca1—C1^iii^	55.22 (6)	Cs1^viii^—O4—Cs1^vi^	120.35 (6)
O3—Ca1—C1^iii^	91.35 (7)	C2—O3—Ca1	115.54 (14)
O1—Ca1—C1^iii^	146.48 (7)	C2—O3—Cs1	138.80 (14)
O1^iii^—Ca1—C1^iii^	23.78 (6)	Ca1—O3—Cs1	90.28 (6)
O2W^iii^—Ca1—C1	115.59 (7)	Cs1—O1W—H1	95 (4)
O2W—Ca1—C1	95.70 (7)	Cs1—O1W—H2	141 (4)
O3^iii^—Ca1—C1	91.35 (7)	H1—O1W—H2	111 (5)
O3—Ca1—C1	55.22 (6)	Ca1—O2W—Cs1	95.21 (6)
O1—Ca1—C1	23.78 (6)	Ca1—O2W—H3	126 (3)
O1^iii^—Ca1—C1	146.48 (7)	Cs1—O2W—H3	105 (3)
C1^iii^—Ca1—C1	134.14 (9)	Ca1—O2W—H4	116 (4)
O2W^iii^—Ca1—C2^iii^	91.60 (7)	Cs1—O2W—H4	106 (4)
O2W—Ca1—C2^iii^	147.17 (7)	H3—O2W—H4	106 (5)
			
O2^i^—Cs1—Ca1—O2W^iii^	115.19 (8)	O2—C1—O1—Cs1^vii^	55.7 (3)
O4^ii^—Cs1—Ca1—O2W^iii^	39.97 (10)	C2—C1—O1—Cs1^vii^	−125.96 (15)
O1W—Cs1—Ca1—O2W^iii^	−79.43 (7)	Ca1—C1—O1—Cs1^vii^	−129.79 (16)
O1^iii^—Cs1—Ca1—O2W^iii^	−63.90 (9)	Cs1^vi^—C1—O1—Cs1^vii^	129.51 (16)
O2^iv^—Cs1—Ca1—O2W^iii^	−149.93 (7)	O2W^iii^—Ca1—O1—C1	175.89 (16)
O1^v^—Cs1—Ca1—O2W^iii^	0.17 (7)	O2W—Ca1—O1—C1	−89.85 (17)
O2W—Cs1—Ca1—O2W^iii^	61.95 (13)	O3^iii^—Ca1—O1—C1	87.52 (16)
O4^iv^—Cs1—Ca1—O2W^iii^	−135.51 (8)	O3—Ca1—O1—C1	−2.85 (16)
O3—Cs1—Ca1—O2W^iii^	174.67 (9)	O1^iii^—Ca1—O1—C1	42.85 (15)
C1^iv^—Cs1—Ca1—O2W^iii^	−134.13 (7)	C1^iii^—Ca1—O1—C1	72.6 (2)
C1^v^—Cs1—Ca1—O2W^iii^	18.20 (7)	C2^iii^—Ca1—O1—C1	83.21 (17)
C2^iv^—Cs1—Ca1—O2W^iii^	−125.01 (8)	C2—Ca1—O1—C1	−2.27 (14)
O2^i^—Cs1—Ca1—O2W	53.23 (7)	Cs1^iii^—Ca1—O1—C1	134.50 (17)
O4^ii^—Cs1—Ca1—O2W	−21.98 (10)	Cs1—Ca1—O1—C1	−39.83 (18)
O1W—Cs1—Ca1—O2W	−141.38 (7)	O2W^iii^—Ca1—O1—Cs1^iii^	41.39 (6)
O1^iii^—Cs1—Ca1—O2W	−125.86 (9)	O2W—Ca1—O1—Cs1^iii^	135.65 (6)
O2^iv^—Cs1—Ca1—O2W	148.11 (7)	O3^iii^—Ca1—O1—Cs1^iii^	−46.97 (6)
O1^v^—Cs1—Ca1—O2W	−61.78 (7)	O3—Ca1—O1—Cs1^iii^	−137.35 (7)
O4^iv^—Cs1—Ca1—O2W	162.53 (8)	O1^iii^—Ca1—O1—Cs1^iii^	−91.7 (5)
O3—Cs1—Ca1—O2W	112.72 (8)	C1^iii^—Ca1—O1—Cs1^iii^	−61.87 (12)
C1^iv^—Cs1—Ca1—O2W	163.92 (7)	C1—Ca1—O1—Cs1^iii^	−134.50 (18)
C1^v^—Cs1—Ca1—O2W	−43.75 (7)	C2^iii^—Ca1—O1—Cs1^iii^	−51.29 (7)
C2^iv^—Cs1—Ca1—O2W	173.04 (7)	C2—Ca1—O1—Cs1^iii^	−136.77 (8)
O2^i^—Cs1—Ca1—O3^iii^	−132.36 (7)	Cs1—Ca1—O1—Cs1^iii^	−174.33 (3)
O4^ii^—Cs1—Ca1—O3^iii^	152.42 (10)	O2W^iii^—Ca1—O1—Cs1^vii^	−58.29 (8)
O1W—Cs1—Ca1—O3^iii^	33.02 (7)	O2W—Ca1—O1—Cs1^vii^	35.97 (8)
O1^iii^—Cs1—Ca1—O3^iii^	48.55 (8)	O3^iii^—Ca1—O1—Cs1^vii^	−146.65 (8)
O2^iv^—Cs1—Ca1—O3^iii^	−37.49 (6)	O3—Ca1—O1—Cs1^vii^	122.98 (8)
O1^v^—Cs1—Ca1—O3^iii^	112.62 (6)	O1^iii^—Ca1—O1—Cs1^vii^	168.7 (4)
O2W—Cs1—Ca1—O3^iii^	174.40 (8)	C1^iii^—Ca1—O1—Cs1^vii^	−161.55 (9)
O4^iv^—Cs1—Ca1—O3^iii^	−23.07 (8)	C1—Ca1—O1—Cs1^vii^	125.83 (19)
O3—Cs1—Ca1—O3^iii^	−72.88 (11)	C2^iii^—Ca1—O1—Cs1^vii^	−150.97 (6)
C1^iv^—Cs1—Ca1—O3^iii^	−21.68 (7)	C2—Ca1—O1—Cs1^vii^	123.55 (10)
C1^v^—Cs1—Ca1—O3^iii^	130.65 (7)	Cs1^iii^—Ca1—O1—Cs1^vii^	−99.68 (7)
C2^iv^—Cs1—Ca1—O3^iii^	−12.56 (7)	Cs1—Ca1—O1—Cs1^vii^	86.00 (7)
O2^i^—Cs1—Ca1—O3	−59.48 (7)	O1—C1—O2—Cs1^i^	−90.7 (4)
O4^ii^—Cs1—Ca1—O3	−134.70 (10)	C2—C1—O2—Cs1^i^	91.0 (3)
O1W—Cs1—Ca1—O3	105.90 (7)	Ca1—C1—O2—Cs1^i^	−106.6 (7)
O1^iii^—Cs1—Ca1—O3	121.43 (8)	Cs1^vi^—C1—O2—Cs1^i^	141.3 (3)
O2^iv^—Cs1—Ca1—O3	35.39 (7)	Cs1^vii^—C1—O2—Cs1^i^	−48.5 (3)
O1^v^—Cs1—Ca1—O3	−174.50 (6)	O1—C1—O2—Cs1^vi^	128.0 (2)
O2W—Cs1—Ca1—O3	−112.72 (8)	C2—C1—O2—Cs1^vi^	−50.3 (2)
O4^iv^—Cs1—Ca1—O3	49.81 (8)	Ca1—C1—O2—Cs1^vi^	112.1 (7)
C1^iv^—Cs1—Ca1—O3	51.20 (7)	Cs1^vii^—C1—O2—Cs1^vi^	170.22 (7)
C1^v^—Cs1—Ca1—O3	−156.47 (7)	O2—C1—C2—O4	−3.0 (3)
C2^iv^—Cs1—Ca1—O3	60.32 (7)	O1—C1—C2—O4	178.5 (2)
O2^i^—Cs1—Ca1—O1	−15.86 (8)	Ca1—C1—C2—O4	−178.8 (2)
O4^ii^—Cs1—Ca1—O1	−91.08 (11)	Cs1^vi^—C1—C2—O4	−42.18 (19)
O1W—Cs1—Ca1—O1	149.52 (7)	Cs1^vii^—C1—C2—O4	114.1 (2)
O1^iii^—Cs1—Ca1—O1	165.05 (12)	O2—C1—C2—O3	175.6 (2)
O2^iv^—Cs1—Ca1—O1	79.01 (7)	O1—C1—C2—O3	−2.9 (3)
O1^v^—Cs1—Ca1—O1	−130.88 (6)	Ca1—C1—C2—O3	−0.25 (16)
O2W—Cs1—Ca1—O1	−69.10 (9)	Cs1^vi^—C1—C2—O3	136.42 (18)
O4^iv^—Cs1—Ca1—O1	93.43 (8)	Cs1^vii^—C1—C2—O3	−67.3 (2)
O3—Cs1—Ca1—O1	43.62 (8)	O2—C1—C2—Ca1	175.9 (2)
C1^iv^—Cs1—Ca1—O1	94.82 (7)	O1—C1—C2—Ca1	−2.63 (16)
C1^v^—Cs1—Ca1—O1	−112.85 (7)	Cs1^vi^—C1—C2—Ca1	136.67 (5)
C2^iv^—Cs1—Ca1—O1	103.94 (7)	Cs1^vii^—C1—C2—Ca1	−67.05 (14)
O2^i^—Cs1—Ca1—O1^iii^	179.09 (7)	O2—C1—C2—Cs1^vi^	39.20 (19)
O4^ii^—Cs1—Ca1—O1^iii^	103.88 (10)	O1—C1—C2—Cs1^vi^	−139.29 (19)
O1W—Cs1—Ca1—O1^iii^	−15.53 (7)	Ca1—C1—C2—Cs1^vi^	−136.67 (5)
O2^iv^—Cs1—Ca1—O1^iii^	−86.03 (7)	Cs1^vii^—C1—C2—Cs1^vi^	156.28 (15)
O1^v^—Cs1—Ca1—O1^iii^	64.08 (8)	O2W^iii^—Ca1—C2—O4	174.0 (7)
O2W—Cs1—Ca1—O1^iii^	125.86 (9)	O2W—Ca1—C2—O4	−86.1 (7)
O4^iv^—Cs1—Ca1—O1^iii^	−71.61 (8)	O3^iii^—Ca1—C2—O4	85.2 (7)
O3—Cs1—Ca1—O1^iii^	−121.43 (8)	O3—Ca1—C2—O4	−4.0 (7)
C1^iv^—Cs1—Ca1—O1^iii^	−70.23 (7)	O1—Ca1—C2—O4	177.4 (8)
C1^v^—Cs1—Ca1—O1^iii^	82.10 (7)	O1^iii^—Ca1—C2—O4	6.8 (7)
C2^iv^—Cs1—Ca1—O1^iii^	−61.11 (7)	C1^iii^—Ca1—C2—O4	32.2 (7)
O2^i^—Cs1—Ca1—C1^iii^	−163.20 (6)	C1—Ca1—C2—O4	175.6 (8)
O4^ii^—Cs1—Ca1—C1^iii^	121.58 (10)	C2^iii^—Ca1—C2—O4	63.4 (7)
O1W—Cs1—Ca1—C1^iii^	2.18 (6)	Cs1^iii^—Ca1—C2—O4	143.9 (7)
O1^iii^—Cs1—Ca1—C1^iii^	17.71 (7)	Cs1—Ca1—C2—O4	−32.6 (7)
O2^iv^—Cs1—Ca1—C1^iii^	−68.32 (6)	O2W^iii^—Ca1—C2—O3	178.02 (16)
O1^v^—Cs1—Ca1—C1^iii^	81.78 (6)	O2W—Ca1—C2—O3	−82.03 (17)
O2W—Cs1—Ca1—C1^iii^	143.56 (8)	O3^iii^—Ca1—C2—O3	89.18 (18)
O4^iv^—Cs1—Ca1—C1^iii^	−53.90 (7)	O1—Ca1—C2—O3	−178.59 (19)
O3—Cs1—Ca1—C1^iii^	−103.72 (7)	O1^iii^—Ca1—C2—O3	10.82 (18)
C1^iv^—Cs1—Ca1—C1^iii^	−52.52 (7)	C1^iii^—Ca1—C2—O3	36.19 (17)
C1^v^—Cs1—Ca1—C1^iii^	99.81 (5)	C1—Ca1—C2—O3	179.7 (2)
C2^iv^—Cs1—Ca1—C1^iii^	−43.40 (6)	C2^iii^—Ca1—C2—O3	67.45 (15)
O2^i^—Cs1—Ca1—C1	−31.54 (6)	Cs1^iii^—Ca1—C2—O3	147.91 (15)
O4^ii^—Cs1—Ca1—C1	−106.76 (10)	Cs1—Ca1—C2—O3	−28.54 (15)
O1W—Cs1—Ca1—C1	133.84 (6)	O2W^iii^—Ca1—C2—C1	−1.64 (19)
O1^iii^—Cs1—Ca1—C1	149.37 (8)	O2W—Ca1—C2—C1	98.31 (12)
O2^iv^—Cs1—Ca1—C1	63.34 (6)	O3^iii^—Ca1—C2—C1	−90.48 (12)
O1^v^—Cs1—Ca1—C1	−146.56 (6)	O3—Ca1—C2—C1	−179.7 (2)
O2W—Cs1—Ca1—C1	−84.78 (8)	O1—Ca1—C2—C1	1.75 (11)
O4^iv^—Cs1—Ca1—C1	77.76 (7)	O1^iii^—Ca1—C2—C1	−168.84 (11)
O3—Cs1—Ca1—C1	27.94 (7)	C1^iii^—Ca1—C2—C1	−143.47 (10)
C1^iv^—Cs1—Ca1—C1	79.14 (4)	C2^iii^—Ca1—C2—C1	−112.21 (12)
C1^v^—Cs1—Ca1—C1	−128.53 (6)	Cs1^iii^—Ca1—C2—C1	−31.75 (11)
C2^iv^—Cs1—Ca1—C1	88.26 (6)	Cs1—Ca1—C2—C1	151.80 (11)
O2^i^—Cs1—Ca1—C2^iii^	−146.25 (6)	O2W^iii^—Ca1—C2—Cs1^vi^	56.78 (16)
O4^ii^—Cs1—Ca1—C2^iii^	138.54 (9)	O2W—Ca1—C2—Cs1^vi^	156.73 (9)
O1W—Cs1—Ca1—C2^iii^	19.14 (6)	O3^iii^—Ca1—C2—Cs1^vi^	−32.05 (9)
O1^iii^—Cs1—Ca1—C2^iii^	34.66 (7)	O3—Ca1—C2—Cs1^vi^	−121.23 (19)
O2^iv^—Cs1—Ca1—C2^iii^	−51.37 (5)	O1—Ca1—C2—Cs1^vi^	60.17 (9)
O1^v^—Cs1—Ca1—C2^iii^	98.74 (5)	O1^iii^—Ca1—C2—Cs1^vi^	−110.41 (9)
O2W—Cs1—Ca1—C2^iii^	160.52 (8)	C1^iii^—Ca1—C2—Cs1^vi^	−85.05 (9)
O4^iv^—Cs1—Ca1—C2^iii^	−36.95 (7)	C1—Ca1—C2—Cs1^vi^	58.42 (11)
O3—Cs1—Ca1—C2^iii^	−86.76 (7)	C2^iii^—Ca1—C2—Cs1^vi^	−53.78 (6)
C1^iv^—Cs1—Ca1—C2^iii^	−35.56 (6)	Cs1^iii^—Ca1—C2—Cs1^vi^	26.67 (8)
C1^v^—Cs1—Ca1—C2^iii^	116.76 (6)	Cs1—Ca1—C2—Cs1^vi^	−149.78 (8)
C2^iv^—Cs1—Ca1—C2^iii^	−26.45 (8)	O3—C2—O4—Cs1^viii^	18.6 (3)
O2^i^—Cs1—Ca1—C2	−46.56 (6)	C1—C2—O4—Cs1^viii^	−163.01 (13)
O4^ii^—Cs1—Ca1—C2	−121.77 (9)	Ca1—C2—O4—Cs1^viii^	21.8 (8)
O1W—Cs1—Ca1—C2	118.83 (6)	Cs1^vi^—C2—O4—Cs1^viii^	146.96 (19)
O1^iii^—Cs1—Ca1—C2	134.35 (7)	O3—C2—O4—Cs1^vi^	−128.4 (2)
O2^iv^—Cs1—Ca1—C2	48.32 (5)	C1—C2—O4—Cs1^vi^	50.0 (2)
O1^v^—Cs1—Ca1—C2	−161.57 (5)	Ca1—C2—O4—Cs1^vi^	−125.2 (7)
O2W—Cs1—Ca1—C2	−99.79 (7)	O4—C2—O3—Ca1	178.86 (19)
O4^iv^—Cs1—Ca1—C2	62.74 (7)	C1—C2—O3—Ca1	0.4 (2)
O3—Cs1—Ca1—C2	12.93 (7)	Cs1^vi^—C2—O3—Ca1	98.19 (19)
C1^iv^—Cs1—Ca1—C2	64.13 (6)	O4—C2—O3—Cs1	−57.5 (3)
C1^v^—Cs1—Ca1—C2	−143.54 (6)	C1—C2—O3—Cs1	123.99 (18)
C2^iv^—Cs1—Ca1—C2	73.24 (2)	Ca1—C2—O3—Cs1	123.6 (2)
O2W^iii^—Ca1—C1—O2	15.1 (8)	Cs1^vi^—C2—O3—Cs1	−138.18 (13)
O2W—Ca1—C1—O2	112.3 (7)	O2W—Ca1—O3—C2	97.94 (16)
O3^iii^—Ca1—C1—O2	−73.7 (8)	O3^iii^—Ca1—O3—C2	−91.27 (16)
O3—Ca1—C1—O2	−163.8 (8)	O1—Ca1—O3—C2	1.18 (16)
O1—Ca1—C1—O2	19.6 (7)	O1^iii^—Ca1—O3—C2	−170.26 (16)
O1^iii^—Ca1—C1—O2	−145.6 (7)	C1^iii^—Ca1—O3—C2	−146.50 (16)
C1^iii^—Ca1—C1—O2	−113.1 (8)	C1—Ca1—O3—C2	−0.22 (14)
C2^iii^—Ca1—C1—O2	−87.4 (7)	C2^iii^—Ca1—O3—C2	−114.89 (16)
C2—Ca1—C1—O2	−164.0 (8)	Cs1^iii^—Ca1—O3—C2	−36.38 (17)
Cs1^iii^—Ca1—C1—O2	−17.4 (7)	Cs1—Ca1—O3—C2	146.74 (17)
Cs1—Ca1—C1—O2	167.0 (7)	O2W—Ca1—O3—Cs1	−48.80 (6)
O2W^iii^—Ca1—C1—O1	−4.56 (18)	O3^iii^—Ca1—O3—Cs1	121.99 (6)
O2W—Ca1—C1—O1	92.68 (17)	O1—Ca1—O3—Cs1	−145.56 (6)
O3^iii^—Ca1—C1—O1	−93.30 (16)	O1^iii^—Ca1—O3—Cs1	43.00 (6)
O3—Ca1—C1—O1	176.60 (19)	C1^iii^—Ca1—O3—Cs1	66.76 (5)
O1^iii^—Ca1—C1—O1	−165.18 (13)	C1—Ca1—O3—Cs1	−146.96 (8)
C1^iii^—Ca1—C1—O1	−132.75 (17)	C2^iii^—Ca1—O3—Cs1	98.37 (5)
C2^iii^—Ca1—C1—O1	−106.99 (16)	C2—Ca1—O3—Cs1	−146.74 (17)
C2—Ca1—C1—O1	176.4 (2)	Cs1^iii^—Ca1—O3—Cs1	176.877 (14)
Cs1^iii^—Ca1—C1—O1	−37.03 (15)	O2^i^—Cs1—O3—C2	−19.6 (2)
Cs1—Ca1—C1—O1	147.37 (15)	O4^ii^—Cs1—O3—C2	−3.0 (3)
O2W^iii^—Ca1—C1—C2	179.01 (11)	O1W—Cs1—O3—C2	150.9 (2)
O2W—Ca1—C1—C2	−83.74 (12)	O1^iii^—Cs1—O3—C2	−164.4 (2)
O3^iii^—Ca1—C1—C2	90.27 (12)	O2^iv^—Cs1—O3—C2	85.8 (2)
O3—Ca1—C1—C2	0.17 (11)	O1^v^—Cs1—O3—C2	−124.3 (2)
O1—Ca1—C1—C2	−176.4 (2)	O2W—Cs1—O3—C2	−94.1 (2)
O1^iii^—Ca1—C1—C2	18.39 (18)	O4^iv^—Cs1—O3—C2	79.9 (2)
C1^iii^—Ca1—C1—C2	50.82 (10)	C1^iv^—Cs1—O3—C2	97.4 (2)
C2^iii^—Ca1—C1—C2	76.58 (14)	C1^v^—Cs1—O3—C2	−101.0 (2)
Cs1^iii^—Ca1—C1—C2	146.54 (11)	C2^iv^—Cs1—O3—C2	95.3 (2)
Cs1—Ca1—C1—C2	−29.06 (11)	O2^i^—Cs1—O3—Ca1	111.65 (7)
O2W^iii^—Ca1—C1—Cs1^vi^	117.19 (10)	O4^ii^—Cs1—O3—Ca1	128.25 (11)
O2W—Ca1—C1—Cs1^vi^	−145.57 (10)	O1W—Cs1—O3—Ca1	−77.83 (6)
O3^iii^—Ca1—C1—Cs1^vi^	28.45 (10)	O1^iii^—Cs1—O3—Ca1	−33.05 (5)
O3—Ca1—C1—Cs1^vi^	−61.65 (9)	O2^iv^—Cs1—O3—Ca1	−142.87 (7)
O1—Ca1—C1—Cs1^vi^	121.8 (2)	O1^v^—Cs1—O3—Ca1	6.97 (8)
O1^iii^—Ca1—C1—Cs1^vi^	−43.43 (17)	O2W—Cs1—O3—Ca1	37.23 (5)
C1^iii^—Ca1—C1—Cs1^vi^	−11.00 (6)	O4^iv^—Cs1—O3—Ca1	−148.82 (5)
C2^iii^—Ca1—C1—Cs1^vi^	14.76 (11)	C1^iv^—Cs1—O3—Ca1	−131.32 (6)
C2—Ca1—C1—Cs1^vi^	−61.82 (11)	C1^v^—Cs1—O3—Ca1	30.32 (8)
Cs1^iii^—Ca1—C1—Cs1^vi^	84.72 (8)	C2^iv^—Cs1—O3—Ca1	−133.39 (5)
Cs1—Ca1—C1—Cs1^vi^	−90.88 (8)	O2W^iii^—Ca1—O2W—Cs1	−136.80 (8)
O2W^iii^—Ca1—C1—Cs1^vii^	−43.40 (8)	O3—Ca1—O2W—Cs1	52.16 (6)
O2W—Ca1—C1—Cs1^vii^	53.84 (7)	O1—Ca1—O2W—Cs1	130.87 (6)
O3^iii^—Ca1—C1—Cs1^vii^	−132.14 (6)	O1^iii^—Ca1—O2W—Cs1	−40.35 (6)
O3—Ca1—C1—Cs1^vii^	137.76 (8)	C1^iii^—Ca1—O2W—Cs1	−38.51 (8)
O1—Ca1—C1—Cs1^vii^	−38.84 (15)	C1—Ca1—O2W—Cs1	106.96 (6)
O1^iii^—Ca1—C1—Cs1^vii^	155.98 (10)	C2^iii^—Ca1—O2W—Cs1	−37.56 (14)
C1^iii^—Ca1—C1—Cs1^vii^	−171.59 (5)	C2—Ca1—O2W—Cs1	75.56 (5)
C2^iii^—Ca1—C1—Cs1^vii^	−145.83 (5)	Cs1^iii^—Ca1—O2W—Cs1	177.031 (16)
C2—Ca1—C1—Cs1^vii^	137.59 (12)	O2^i^—Cs1—O2W—Ca1	−117.98 (8)
Cs1^iii^—Ca1—C1—Cs1^vii^	−75.87 (3)	O4^ii^—Cs1—O2W—Ca1	169.09 (5)
Cs1—Ca1—C1—Cs1^vii^	108.53 (3)	O1W—Cs1—O2W—Ca1	43.91 (7)
O2—C1—O1—Ca1	−174.53 (19)	O1^iii^—Cs1—O2W—Ca1	30.93 (5)
C2—C1—O1—Ca1	3.8 (2)	O2^iv^—Cs1—O2W—Ca1	−38.25 (8)
Cs1^vi^—C1—O1—Ca1	−100.7 (2)	O1^v^—Cs1—O2W—Ca1	118.48 (6)
Cs1^vii^—C1—O1—Ca1	129.79 (16)	O4^iv^—Cs1—O2W—Ca1	−66.5 (2)
O2—C1—O1—Cs1^iii^	−55.6 (3)	O3—Cs1—O2W—Ca1	−38.14 (5)
C2—C1—O1—Cs1^iii^	122.78 (17)	C1^iv^—Cs1—O2W—Ca1	−21.98 (9)
Ca1—C1—O1—Cs1^iii^	118.94 (19)	C1^v^—Cs1—O2W—Ca1	136.42 (7)
Cs1^vi^—C1—O1—Cs1^iii^	18.2 (3)	C2^iv^—Cs1—O2W—Ca1	−14.46 (15)
Cs1^vii^—C1—O1—Cs1^iii^	−111.26 (16)		

Symmetry codes: (i) −*x*+1/2, −*y*+1/2, −*z*+1; (ii) −*x*+1/2, *y*+1/2, −*z*+1/2; (iii) −*x*, *y*, −*z*+1/2; (iv) *x*, −*y*, *z*−1/2; (v) *x*, −*y*+1, *z*−1/2; (vi) *x*, −*y*, *z*+1/2; (vii) *x*, −*y*+1, *z*+1/2; (viii) −*x*+1/2, *y*−1/2, −*z*+1/2.

### Hydrogen-bond geometry (Å, º)

**Table:**

*D*—H···*A*	*D*—H	H···*A*	*D*···*A*	*D*—H···*A*
O1*W*—H1···O2^iii^	0.81 (7)	1.91 (7)	2.714 (4)	172 (6)
O1*W*—H2···O3^ix^	0.81 (4)	1.95 (4)	2.736 (2)	163 (7)
O2*W*—H3···O1*W*^x^	0.82 (3)	1.89 (3)	2.680 (2)	164 (5)
O2*W*—H4···O4^i^	0.81 (4)	1.92 (3)	2.724 (3)	176 (7)

Symmetry codes: (i) −*x*+1/2, −*y*+1/2, −*z*+1; (iii) −*x*, *y*, −*z*+1/2; (ix) −*x*, −*y*, −*z*; (x) −*x*, −*y*+1, −*z*.
